# Improving the accuracy of gastrointestinal neuroendocrine tumor grading with deep learning

**DOI:** 10.1038/s41598-020-67880-z

**Published:** 2020-07-06

**Authors:** Darshana Govind, Kuang-Yu Jen, Karen Matsukuma, Guofeng Gao, Kristin A. Olson, Dorina Gui, Gregory. E. Wilding, Samuel P. Border, Pinaki Sarder

**Affiliations:** 1 Department of Pathology and Anatomical Sciences, The State University of New York at Buffalo, 955 Main Street, Buffalo, NY 14203 USA; 2 Department of Pathology and Laboratory Medicine, University of California At Davis School of Medicine, Sacramento, CA USA; 3 Department of Biostatistics, The State University of New York, 3435 Main Street, Buffalo, NY 14214 USA

**Keywords:** Gastrointestinal cancer, Machine learning, Biomedical engineering

## Abstract

The Ki-67 index is an established prognostic factor in gastrointestinal neuroendocrine tumors (GI-NETs) and defines tumor grade. It is currently estimated by microscopically examining tumor tissue single-immunostained (SS) for Ki-67 and counting the number of Ki-67-positive and Ki-67-negative tumor cells within a subjectively picked hot-spot. Intraobserver variability in this procedure as well as difficulty in distinguishing tumor from non-tumor cells can lead to inaccurate Ki-67 indices and possibly incorrect tumor grades. We introduce two computational tools that utilize Ki-67 and synaptophysin double-immunostained (DS) slides to improve the accuracy of Ki-67 index quantitation in GI-NETs: (1) Synaptophysin-KI-Estimator (SKIE), a pipeline automating Ki-67 index quantitation via whole-slide image (WSI) analysis and (2) deep-SKIE, a deep learner-based approach where a Ki-67 index heatmap is generated throughout the tumor. Ki-67 indices for 50 GI-NETs were quantitated using SKIE and compared with DS slide assessments by three pathologists using a microscope and a fourth pathologist via manually ticking off each cell, the latter of which was deemed the gold standard (GS). Compared to the GS, SKIE achieved a grading accuracy of 90% and substantial agreement (linear-weighted Cohen’s kappa 0.62). Using DS WSIs, deep-SKIE displayed a training, validation, and testing accuracy of 98.4%, 90.9%, and 91.0%, respectively, significantly higher than using SS WSIs. Since DS slides are not standard clinical practice, we also integrated a cycle generative adversarial network into our pipeline to transform SS into DS WSIs. The proposed methods can improve accuracy and potentially save a significant amount of time if implemented into clinical practice.

## Introduction

The Ki-67 index is an important prognostic marker and the most widely used parameter for grading gastrointestinal neuroendocrine tumors (GI-NETs)^1–3^. The current practice for obtaining the Ki-67 index involves microscopic examination of tumor tissue that is immunostained for only Ki-67 (henceforth referred to as single-immunostained or SS). First, a hot-spot (tumor region with the highest density of Ki-67-positive tumor cells) is selected, which is then used to manually obtain the percentage of Ki-67-positive tumor cells by counting a total of 500 to 2000 tumor cells^2,3^. Current GI-NET grading, as proposed by the World Health Organization (WHO) 2017 recommendations^4,5^ is based entirely on the mitotic count and Ki-67 index, of which the latter has proven to more accurately reflect biological behavior^6,7^. A Ki-67 index of < 3% is grade 1 (G1), between 3 and 20% is grade 2 (G2), and > 20% is grade 3 (G3)^4,5^. Nevertheless, the Ki-67 index still suffers from intra- and inter-observer variability^8^, especially for differentiating G1 from G2 GI-NETs, given the subjective nature of hot-spot selection as well as the common practice of “eyeball” estimation among pathologists due to the cumbersome process of manually counting individual tumor cells^9^. Thus, an automated method of quantifying the Ki-67 index, especially one that is capable of accurately differentiating G1 from G2 GI-NETs, that is rapid, reliable, and robust would greatly improve GI-NET grading as well as provide increased efficiency to workflow.

Another major limitation in the assessment of the Ki-67 index is the accidental inclusion of proliferating non-tumor cells within the tumor sample^10^. For GI-NETs in particular, intratumoral endothelial cells, background epithelium (e.g., glands, crypts), and lymphocytes can be Ki-67 positive. These Ki-67-positive non-neoplastic cells, if included in the analysis, may introduce error in estimating the Ki-67 index^10^, thereby the estimated tumor grade possibly becomes error-prone. The difficulty in distinguishing tumor from non-tumor cells on standard immunostained sections also makes the assessment of the Ki-67 index prone to inconsistency between pathologists.

Several computational methods have emerged in the recent years to extract Ki-67 indices^11–16^. Shi et al. developed an automated method for the computation of Ki-67 index in nasopharyngeal carcinoma via smoothing, K-means clustering, and feature extraction^11^. Zhong et al. computed the Ki-67 index in primary invasive breast cancer images^12^. Swiderka et al. used morphological techniques and texture analysis to separate hemorrhagic areas and background from tumor regions within whole slide images (WSIs)^13^. Konsti et al. reported virtual application for Ki-67 assessment in breast cancer primarily using the ImageJ software^14^. The WSIs were separated into hematoxylin and diaminobenzidine (DAB) channels using a color deconvolution plugin. Xing et al. developed a machine learning method to compute the Ki-67 index in neuroendocrine tumor images, wherein pathologist annotated hot-spots were used to assess the Ki-67 index^15^. Nielsen et al*.* have demonstrated the use of a mixture of immunostains to automate Ki-67 index quantitation in melanocytic lesions^16^. In practice, many pathologists use ImmunoRatio, a publicly available web application that generates an automated quantification of the Ki-67 index based on a pathologist-selected tumor image and a pathologist-selected tumor nuclear size^17^. However, all of the above-mentioned methods either do not specifically distinguish between neoplastic and non-neoplastic cells, require manual selection of hot-spots (which is subjective and error prone), or lack scalability of the algorithms (which reduces their reproducibility and robustness).

**Figure 1:**
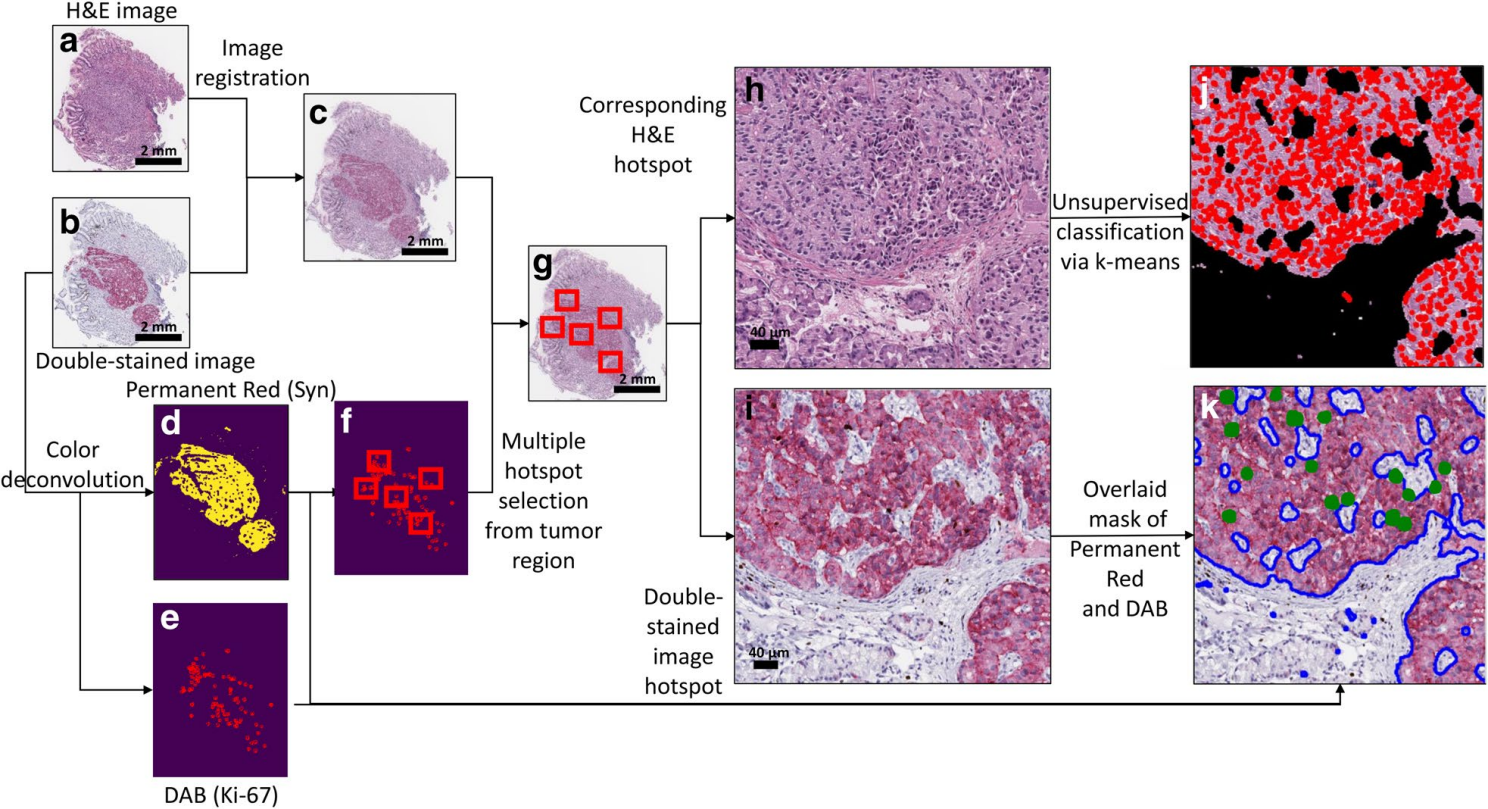
Schematic diagram for Synaptophyin-Ki-67 Index Estimator (SKIE). (**a**) Whole-slide image (WSI) of gastrointestinal neuroendocrine tumor tissue section stained with hematoxylin and eosin (H&E). (**b**) WSI of the adjacent tissue section stained with synaptophysin (red) and Ki-67 (brown) (or DS WSI). (**c**) Result of image registration by matching manually selected landmarks within 1a and 1b. (**d**, **e**) Binary mask of synaptophysin positive region and Ki-67 positive cells, respectively, obtained upon color deconvolution and morphological processing. (**f**) Automated detection of five candidate hot-spots containing the highest density of Ki-67 positive cells within tumor regions. (**g**) Selected candidate hot-spots chosen from the registered images. (**h**, **i**) Extracted hot-spots from the H&E and the double-immunostained images, respectively. (**j**) Overlay of nuclei mask obtained via unsupervised classification of pixels via k-means clustering of (**h**) to obtain all cells within tumor regions. (**k**) Overlay of the hot-spot using masks from (**d**) and (**e**) to obtain Ki-67 positive cells (highlighted in green) within tumor regions (bounded in blue).

**Figure 2:**
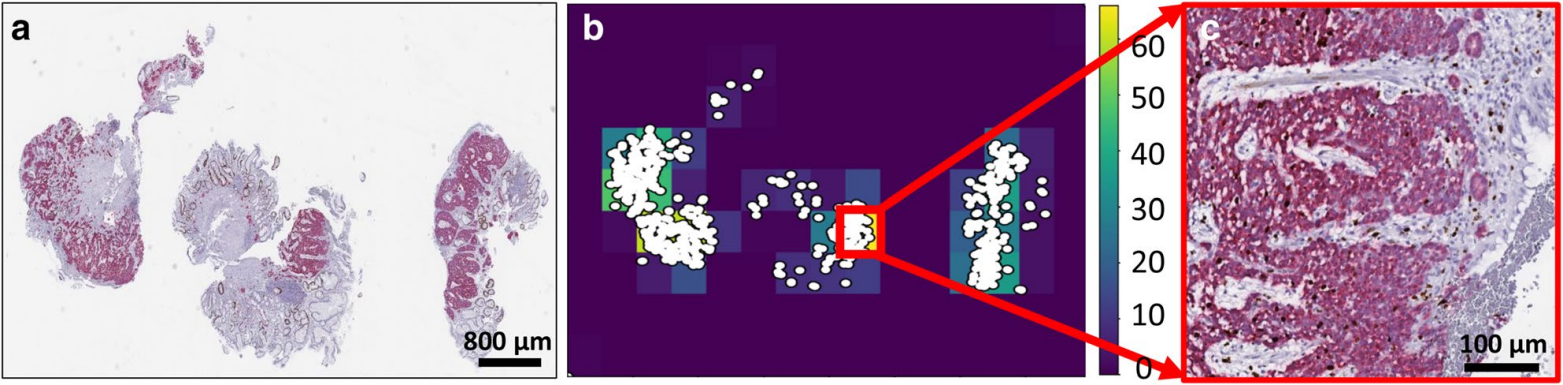
Automated hot-spot detection. (**a**) Double-immunostained whole slide image (DS WSI) with (**b**) corresponding heat-map of 2D histogram of the binary mask indicating location of Ki-67 positive nuclei (white dots). (**c**) Highest density region in (**b**) extracted in high resolution.

**Figure 3:**
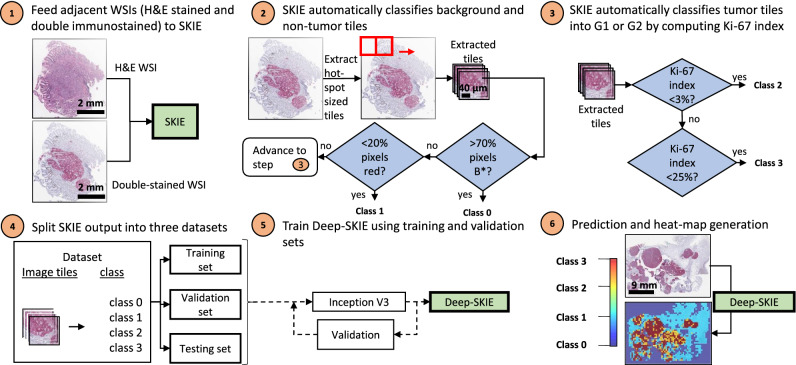
Deep-SKIE computational pipeline. In step 1, the DS and adjacent H&E WSI are fed to SKIE. In steps 2 and 3, SKIE classifies hot-spot-sized tiles from the WSI into class 0 (> 70% background (B* in step 2)), class 1 (< 20% synaptophysin), class 2 (G1) or class 3 (G2). In steps 4 and 5, the extracted tiles and labels are used to train, validate, and test the Inception V3 network. In step 6, the test set predictions are displayed as a heat-map highlighting the tumor distribution across the WSI.

The goal of this study is to bridge the above gaps via machine learning, and to improve the accuracy of current GI-NET grading. To achieve this goal, we developed two automated computational pipelines for GI-NET grading based on analysis of WSIs double-immunostained (DS) for synaptophysin (a marker for NETs) and Ki-67^10^. First, we developed an integrated approach termed Synaptophyin-Ki-67 Index Estimator (SKIE) (Fig. 1), in which DS WSIs along with their adjacent hematoxylin and eosin (H&E)-stained sections were computationally analyzed to locate tumor cells, automatically detect hot-spots (Fig. 2), and calculate the Ki-67 index from those hot-spots. Ki-67 indices as well as tumor grades assigned by SKIE were compared to the results of three gastrointestinal pathologists as well as a fourth gold standard (GS) pathologist, the latter of which was based on exhaustive manual counting of camera-captured hot-spot images. Second, we developed deep-SKIE (Fig. 3), a deep learner-based pipeline which classifies each hot-spot-sized tile in a WSI into one of four classes: background, non-tumor, G1 tumor, and G2 tumor. When trained and tested on DS WSIs, deep-SKIE generated a higher classification accuracy than the SS WSIs, thereby demonstrating the importance of DS WSIs when compared to the standard SS WSIs. While SKIE automates the current clinical practice of grading a tissue based on the Ki-67 index estimated from a hot-spot; deep-SKIE, in contrast, generates a holistic view of the tumor via a Ki-67 index-based heatmap. Lastly, since DS slides are not standard clinical practice, we developed a cycle generative adversarial network^18^ (GAN)-based pipeline to transform SS WSIs into DS WSIs. Cycle GAN is a cutting-edge computational machine learning tool that transforms images from one domain to another. For example, one can train this algorithm with a set of horse images and a set of zebra images, and cycle GAN can learn to transfer a horse image to be a zebra image and vice versa*.* For the purposes of this study, we were able to produce virtual DS WSIs from SS WSIs. The cycle GAN-generated virtual DS WSIs were processed through SKIE and deep-SKIE, which generated comparable results to that of the actual DS WSIs. For this study, we focused on G1 and G2 cases of GI-NETs given that grading these tumors clinically are the most challenging.

## Results

### SKIE-based tumor grade and Ki-67 index performance evaluation

The three pathologists and the GS unanimously agreed upon tumor grades for 38 of the 50 cases (76%); 34 out of 38 (89.5%) of these cases were accurately graded by SKIE. One discrepant case was attributed to section artifacts, including prominent staining inconsistencies and tissue folds. Another discrepant case had no available hot-spot windows containing at least 500 tumor cells. In the other two discrepant cases, SKIE selected a hot-spot with a higher Ki-67 index (i.e. better hot-spot) and therefore assigned a higher grade. The remaining 12 GI-NETs had varying grades assigned by different pathologists; thus, the GS was chosen for comparison. Tumor grades by SKIE for 11 out of these 12 cases (91.7%) matched with the GS. The one discrepant case was assigned a higher grade than the GS, again due to better hot-spot selection by SKIE. SKIE roughly needed 1.4 ± 0.47 s to compute the Ki-67 index from a single hot-spot, while the GS pathologist required approximately 10–15 min per hot-spot.

To summarize, overall when compared to the GS, SKIE agreed with the GS tumor grade in 45 out of 50 cases (90%) and had a Ki-67 index error (defined as the absolute difference between the GS index and that estimated by SKIE) of 0.84 ± 1.02%. If cases with technical issues on slide preparation or cases with insufficient tumor nuclei in SKIE hot-spot windows were excluded, then SKIE agreed with the GS tumor grade in 45 out of 48 cases (93.8%); all three discrepant cases were attributed to SKIE picking better hot-spots with a higher Ki-67 index than the GS.

To verify that SKIE was not overestimating the Ki-67 index, we processed the GS pathologist-picked hot-spots using SKIE on the three cases where SKIE had picked a better hot-spot (Table 1). Based on the exact same hot-spot field, all three tumor grades estimated by SKIE matched with the GS with an index error of 0.24 ± 0.19%. To further verify that SKIE was indeed locating better hot-spots, we extracted the SKIE-picked hot-spots for the three cases with discrepant tumor grades discussed above and had the GS pathologist grade them by exhaustive manual counting (Table 1). The grades for all three cases were rectified by the pathologist to be G2 instead of G1 (as was initially graded based on the pathologist-picked hot-spot), indicating that the SKIE-picked hot-spot was superior to the pathologist-picked hot-spot and better reflected the true tumor grade. Even for cases other than the three mentioned here, SKIE generally picked hot-spots with a higher index than the GS pathologist (36 out of the 50 cases).

**Table 1:**
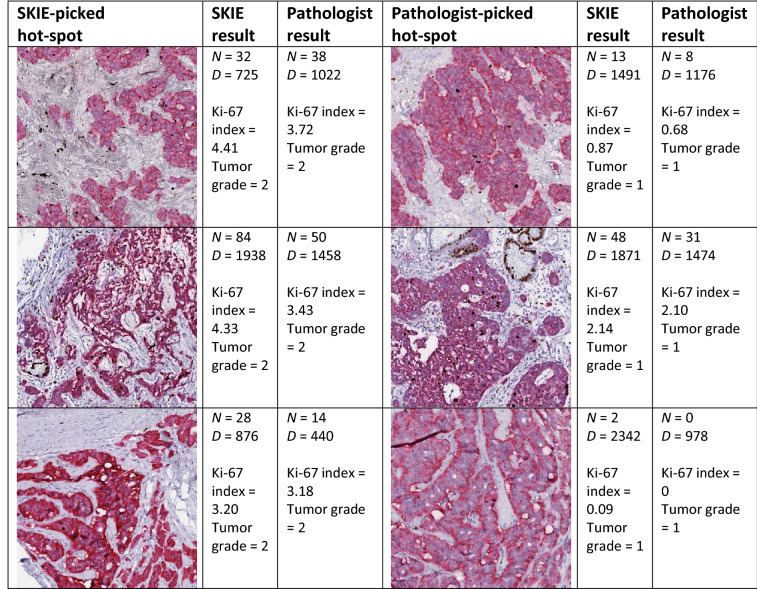
Comparison of SKIE-picked hot-spot versus pathologist-picked hot-spot

The three discrepant cases between SKIE and the GS are shown. The SKIE-picked hot-spot (column 1) is shown with its corresponding results by SKIE (column 2) and the GS pathologist (column 3). The pathologist-picked hot-spot (column 4) is shown with its corresponding results by SKIE (column 5) and the GS pathologist (column 6) [*N*, numerator (Ki-67-positive tumor cells); *D*, denominator (total tumor cells)].

### Inter- and intra-user variation of landmark selection for image registration

The selection of appropriate landmarks is crucial to ensure proper registration of the WSIs. Image registration is an image processing method^19^ to align images of the same context. In this work, images of the same field from adjacent tissue sections stained with different stains were aligned using manually selected landmarks. To test the variation of the computed Ki-67 index due to inter- and intra-user variations in landmark selection, two WSI cases that were close to the border of G1 and G2 with a Ki-67 index in and around the range of 3 ± 0.5% were analyzed to test the robustness of SKIE. One of the co-authors (who did not select the original landmarks (selected by User 1) used for analysis) selected 10 random landmark points on the WSIs, at two independent time points (User 2 and User 3 in Supplementary Fig. S1), which were recorded and compared with the original set of landmarks for analysis. In both cases, the automatically selected hot-spots by SKIE were the same, regardless of registration. As a result, there was no change in tumor grade based on variations in landmark selection for registration (Supplementary Fig. S1).

### Same hot-spot analysis: SKIE versus pathologist

To test the accuracy of SKIE within a selected hot-spot, the exact same hot-spot field (one hot-spot per case) which was chosen and graded by the GS pathologist by exhaustive manual counting was analyzed by SKIE and the generated Ki-67 indices were compared with the GS pathologist’s results. Forty-eight (96%) of the tumor grades matched, among which the average Ki-67 index error was 0.56 ± 1.11%. Out of the two discrepant cases, one was assigned a lower grade than the pathologist because SKIE detected more Ki-67-negative tumor nuclei than the GS pathologist (1,354 versus 770) while the number of tabulated Ki-67-positive nuclei were comparable (27 versus 28). The second case was also assigned a lower grade by SKIE, but in this instance SKIE counted a lower number of Ki-67-positive tumor cells (30 versus 40) but a higher number of total tumor cells (1,320 versus 992), resulting in a Ki-67 index of 2.3% (G1) for SKIE and 4.03% (G2) for the pathologist.

### Same hot-spot analysis: SKIE versus ImmunoRatio

**Figure 4:**
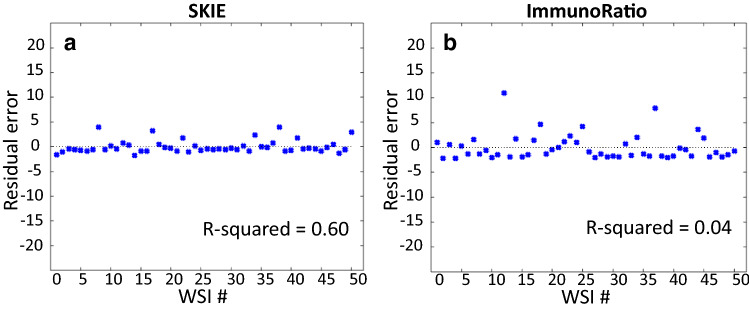
Residual error analysis comparison between SKIE and ImmunoRatio. (**a**) The residual error for SKIE versus (**b**) ImmunoRatio on the same fields as compared to the gold standard of exhaustive manual counting of the pathologist selected hot-spot.

The performance of SKIE was compared to the current most widely used automated immunostain quantification tool, ImmunoRatio^17^ (an ImageJ plugin). This tool is popularly used by pathologists for the quantification of Ki-67 positive nuclei in immunostained tissue sections. Since ImmunoRatio can only be used on standard SS images, each SKIE-picked hot-spot from the DS WSI was located on the adjacent SS WSIs and these latter images were analyzed by ImmunoRatio. SKIE outperformed ImmunoRatio by approximately 2.5-fold (Fig. 4). Supplementary Table S1 shows the comparison of SKIE to ImmunoRatio, highlighting the advantages of SKIE, primarily the automated WSI analysis, hot-spot detection and lower error rate. The high error rate of Ki-67 index as estimated by ImmunoRatio was especially evident in hot-spots wherein several non-tumor Ki-67 positive cells were present (Supplementary Fig. S2).

**Figure 5:**
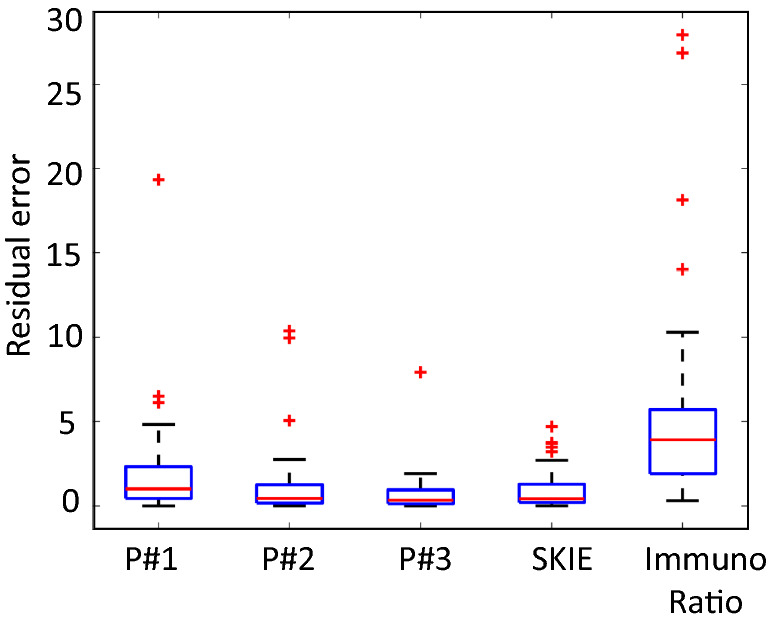
Ki-67 index residual error for participating pathologists, SKIE, and ImmunoRatio compared to exhaustive manual counting. Boxplots show the error rate of SKIE compared to three pathologists (P1, P2, and P3) and that of ImmunoRatio as compared to the gold standard of exhaustive manual counting.

The residual error plot of the three pathologists, SKIE, and ImmunoRatio in comparison to the GS is shown in Fig. 5. SKIE generated comparable results with that of the pathologists’, while being more reproducible, faster, and having a lower index error. Residual error here suggests the difference between the respective Ki-67 index values estimated by the pathologists, SKIE, or ImmunoRatio and by the GS.

### Inter-annotator agreement

The linear weighted Cohen’s kappa for inter-annotator agreement between each of the three pathologists and GS were fair (*κ* = 0.32 with 95% confidence interval (CI) [0.04, 0.60]), substantial (*κ* = 0.67 with CI [0.38, 0.96]), and substantial (*κ* = 0.78 with CI [0.48, 1]) respectively, for tumor grade. When the Ki-67 indices were averaged among the three pathologists and translated to tumor grade, the agreement with GS tumor grade remained at a substantial Cohen’s kappa of 0.67 with CI [0.38, 0.96]. The performance of a baseline classifier, which performs random predictions with a probability equal to the sample distribution (in this case G1 and G2), had a Cohen’s kappa of zero, which indicates that there is no agreement over random chance. The performance of SKIE showed a substantial Cohen’s kappa of 0.62 with CI [0.32, 0.91] (Supplementary Table S2) and for the exact same hot-spot analysis (i.e. the hot-spots picked by GS), SKIE showed a substantial agreement with GS with Cohen’s kappa of 0.73 [0.38, 1]. As further validation, the conditional probability of grade assignment with respect to the GS chosen grade (Supplementary Table S3) showed an overall agreement of 90% between SKIE and the GS.

### Deep-SKIE: tumor heatmap generation via deep learning

Two different deep learners (collectively called deep-SKIE) were trained to predict background, non-tumor, G1 tumor and G2 tumor from tiles extracted from DS WSI (model_SK) and SS WSI (model_K). As expected, model_SK displayed higher accuracy (training accuracy: 98.37%, validation accuracy: 90.90%, and testing accuracy: 90.98%) when compared to model_K (training accuracy: 95.9%, validation accuracy: 86.9%, and testing accuracy: 84.84%). These results depict the improvement in tumor grading accuracy with DS WSIs when compared to SS WSIs.

### Virtual double-immunostains generated from cycle GAN

**Figure 6:**
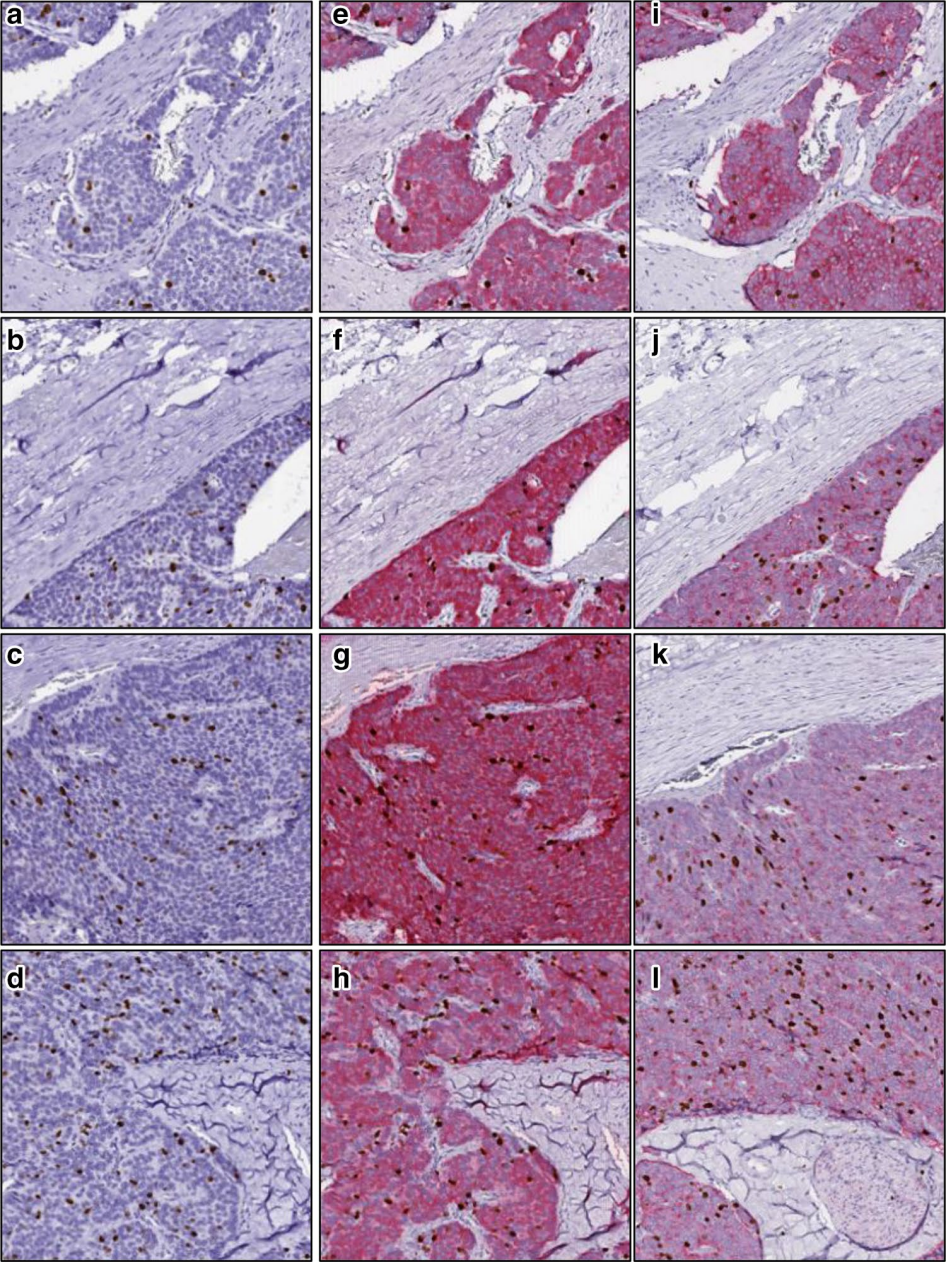
Comparison of cycle GAN-predicted DS tiles with original adjacent DS tiles. (**a**–**d**) A few image tiles from the SS WSI are shown. (**e**–**h**) shows the respective DS tiles as predicted by the cycle GAN using a-d as inputs. (**i**–**l**) shows the original adjacent DS tiles.

Since the double-immunostain technique is not standard clinical practice, we then trained a cycle GAN to automatically generate virtual DS WSIs from standard SS WSIs. After the cycle GAN model was trained using image tiles extracted from ~ 85% of WSIs from both SS and DS WSIs, we tested the model on the remaining 6 hold-out WSIs (five G1 cases and G2 case). These 6 hold-out cases, which were originally SS WSIs, were transformed into virtual DS WSIs by the cycle GAN. Some of the virtual DS tiles, as generated by the model from these hold-out cases, are shown in Fig. 6. To evaluate the performance of the model, the regions virtually stained with synaptophysin in the predicted images were compared to the actual synaptophysin-stained regions in the original adjacent DS section. The regions aligned with an average sensitivity, specificity and accuracy of 93.32%, 81.07%, and 84.61%.

### Analysis of cycle GAN generated WSIs via SKIE

To analyze the performance of the cycle GAN, the virtual DS WSIs were processed by SKIE and the results were compared to that of the original adjacent DS WSIs. All of the 6 virtual DS WSIs were accurately graded by SKIE with an average Ki-67 index error of 1.56 ± 1.15%, when compared to the GS.

### Analysis of cycle GAN generated WSIs via deep-SKIE

**Figure 7:**
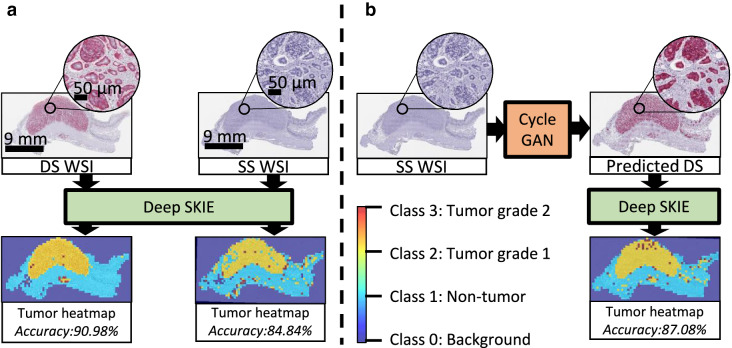
Tumor heatmap prediction. (**a**) Deep-SKIE is tested using a hold-out WSI to predict a heatmap of background, non-tumor, tumor grade 1 and tumor grade 2 in both DS and SS WSI. (**b**) The accuracy of tumor prediction in SS WSI is improved upon transformation into ‘predicted’ DS WSI using the cycle GAN.

The virtual DS WSIs were cropped into hot-spot-sized tiles and fed to deep-SKIE, and the results were compared to that of the original adjacent DS WSIs. The classification accuracy of model_SK was 87.08% when compared to SKIE-generated ground truth, i.e. significantly higher than the SS WSIs (84.84%) (Fig. 7). The results indicate that deep-SKIE along with cycle GANs not only provide additional information to the pathologist by displaying holistic tumor data through a heatmap of local tumor grades based on local Ki-67 indices, but they also improve the Ki-67 index accuracy quantitated on SS images.

## Methods

### Cases, immunohistochemistry, and image acquisition

This study was approved by the Institutional Review Board at the University of California Davis Medical Center. All methods were performed in accordance with the relevant federal guidelines and regulations. Participants were required to be over 18 years of age. Special populations (vulnerable) such as minors, pregnant women, neonates, prisoners, children, and cognitively impaired patients were not included. All patients provided written informed consent, and basic demographic information was collected. All glass slides used in this study were previously produced for a prior study by Matsukuma et al^10^. For each case, the glass slides of the synaptophysin/Ki-67 DS section, the adjacent Ki-67-only-stained or SS section, and the adjacent H&E-stained section (Supplementary Fig. S3) were digitized as WSIs using the Aperio AT2 scanner at 20X magnification (0.50 microns-per-pixel resolution). The SS and DS sections were counterstained with hematoxylin.

### Quantification of Ki-67 index by pathologists

Details on the participating pathologist’s quantification of Ki-67 index were previously described^10^. In this study, the data from the three gastrointestinal pathologists’ individual assessment of the Ki-67 index based on the DS glass slides reviewed under the microscope were used. As a GS, a fourth pathologist independently measured the Ki-67 index by selecting and image capturing one hot-spot on the DS WSI, displaying the image on a monitor, and manually ticking off each Ki-67-positive and negative tumor cell until at least 500 tumor cells were counted. Assessment of the tumor grade was based on the WHO 2017 criteria.

### SKIE: computational pipeline

Figure 1 shows the proposed computational pipeline for SKIE. First, manually selected landmark points were chosen by the user from the H&E (Fig. 1a) and the DS WSI (Fig. 1b) to perform landmark-based image registration^20^ (Fig. 1c). This registration enables the extraction of five candidate hot-spots from the DS WSI and their corresponding H&E fields. The binary masks of the tumor region (Fig. 1d) and the Ki-67-positive nuclei (Fig. 1e) were obtained via color deconvolution^21^ and subsequent morphological processing^19^ of the DS WSIs. Five high-density Ki-67 positive locations (500 × 500 µm^2^) were automatically selected (Fig. 1f) from the tumor region as candidate hot-spots (Fig. 1g), each consisting of a field from the H&E-stained WSI (Fig. 1h) and its corresponding field from the DS (Fig. 1i) WSI. The Ki-67 negative tumor nuclei stained with hematoxylin in the DS images appeared to be obscured in certain locations due to low contrast against Permanent Red (synaptophysin immunostain). To avoid inconsistencies in the detection of Ki-67 negative tumor nuclei due to this poor contrast, the enumeration was performed on the H&E-stained section. Thus, the binary mask of the tumor region was applied to the H&E-stained counterpart of the candidate hot-spot and then the non-proliferating tumor cells were detected via unsupervised classification of pixels using k-means clustering algorithm (with *k* = 3, representing hematoxylin, eosin and the background). The cluster representing hematoxylin was chosen to obtain the mask for all nuclei (highlighted with red contours in Fig. 1j) within the tumor region. The overlap of the tumor region (Fig. 1d) and Ki-67 positive nuclei (Fig. 1e) were extracted to obtain Fig. 1k and ultimately the Ki-67 index from that field.

### Semi-automated image registration

To align the H&E and the DS WSIs, ten corresponding landmark points were selected from both images by the user^22^ (Supplementary Fig. S4). These points were used to compute the similarity transform between the two WSIs, which was used to modify the spatial configurations of the H&E WSI with respect to the DS WSI.

### Detection of tumor regions and Ki-67-positive cells

The color deconvolution algorithm^21^ was applied to separate the two stains (synaptophysin and Ki-67) within the DS WSIs. The boundaries of the Ki-67-positive cells and the synaptophysin positive regions were clearly discernible at ¼th the resolution of the WSI. Therefore, the color deconvolution was performed on the DS WSIs downscaled by a factor of four to reduce the computational complexity. The resultant images were morphologically processed to generate binary masks for the synaptophysin-positive region and the Ki-67-positive cells.

### Hot-spot detection

The binary mask of Ki-67-positive nuclei within the tumor regions was used to detect the hot-spots (Fig. 2a). A 2D histogram was then plotted, from the DS WSI, with the corresponding binning in each dimension to generate a heat-map of Ki-67-positive nuclei (Fig. 2b), thereby identifying high-density regions (Fig. 2c).

### Nuclei clustering via unsupervised pixel classification

Since Ki-67-negative nuclei are more densely spaced than Ki-67-positive nuclei within the tumor regions, the former needed to be analyzed in high resolution. The extracted candidate hot-spots from the H&E counterparts were used for the enumeration of these non-proliferating nuclei (Supplementary Fig. S5). The k-means clustering algorithm was used to segment the candidate hot-spot pixels into three categories: white background, hematoxylin, or eosin^23^. The pixels classified as hematoxylin were extracted and morphologically processed to generate a binary mask of all nuclei.

### Estimation of nucleus counts

Since individual nuclei may be clumped together in some foci, an approximated method for nucleus count was used by computing the ratio between total area of the pixels in the image that were detected as nuclei and the average area of an individual nucleus. The average nucleus area was estimated to be the median of the distribution of the areas of nuclei that are not appeared to be clumped (Supplementary Fig. S6). These single nuclei were detected based on an area threshold applied to the mask of all detected nuclei.

### Ki-67 index and tumor grade

The Ki-67 index was calculated as the percentage of Ki-67-positive tumor nuclei among all tumor nuclei within the hot-spot. The binary masks of Ki-67-positive nuclei and all tumor nuclei were used to generate the Ki-67 index. Tumor grade was assigned based on the WHO 2017 recommendations, with G1 and G2 tumors having a Ki-67 index of < 3% and 3–20%, respectively^2^.

### Statistical analysis

To compare the performance of the SKIE to the GS, linear-weighted Cohen’s kappa^24^ was calculated. Kappa < 0, 0–0.21, 0.21–0.4, 0.41–0.6, 0.61–0.8, and 0.81–1 indicates none, slight, fair, moderate, substantial, and near perfect agreement, respectively. The conditional probability of grade assignment was computed with respect to the GS assigned grades. The 95% confidence intervals of these statistical data were also computed. For the calculation of confidence intervals and Cohen’s kappa, the Clopper–Pearson exact method was used^25^.

### Deep-SKIE for tumor heatmap prediction

WSIs of DS sections for 48 GI-NETs were cropped into hot-spot-sized tiles and categorized into one of four classes using SKIE, which served as ground truth: background (class 0: if the tile has > 70% background pixels) or non-tumor (class 1: < 20% synaptophysin stain), tumor grade 1 (class 2: Ki-67 index < 3%) and tumor grade 2 (class 3: 3% < Ki-67 index < 20%) (Fig. 3). These DS tiles were used to train, validate and test a model we named as ‘model_SK’, for 10 epochs. The corresponding tiles from the registered adjacent SS stained sections were categorized into the same class as their DS counterparts, and were used to train, validate and test a model we named as ‘model_K’. Image tiles which had severe staining artifacts or tissue folds were removed from the test set in both models. The Inception V3 network^26^ was trained and validated using 42 cases (~ 85% of WSIs; 15,232 image tiles) with a batch size of 32 and tested on 6 cases (i.e. 9,436 image tiles), for both models. We used stochastic gradient descent optimization, with an initial learning rate of 0.1, decay rate of 0.01, and momentum of 0.9 for training the weights. Two WSIs with severe staining and technical issues were removed from analysis.

### Cycle GAN based virtual staining

We trained a cycle GAN architecture^18^ to map images from one domain (SS WSI) to another (DS WSI). The training set consisted of ~ 47 K unpaired images of DS and SS tiles obtained from 42 WSIs each. Our model was trained for 10 epochs, on NVIDIA GeForce GTX GPU based on the PyTorch implementation and hyperparameters specified by Zhu et al^18^. Two WSIs with severe staining or technical issues were removed from analysis. The model was tested on the remaining 6 hold-out WSIs. To evaluate the performance of the model, the regions virtually stained with synaptophysin in the predicted images were compared to the regions stained with synaptophysin in the original adjacent DS WSI. The corresponding masks of synaptophysin were compared. The predicted DS WSIs generated by the cycle GAN from these 6 hold-out cases were processed through SKIE and deep-SKIE for comparison with the original DS WSIs.

## Discussion

Accurate assessment of the Ki-67 index is crucial for tumor grading of GI-NETs, which determines the patient’s prognosis and outcome. In this study, we developed two automated computational pipelines, SKIE and deep-SKIE, employing a double-immunostain technique using antibodies against synaptophysin and Ki-67, the former of which is a neuroendocrine marker. SKIE is an integrated approach allowing both automated hot-spot selection and Ki-67 index quantification. The integration of automated hot-spot selection in this study sets it apart from most other studies that have explored automated Ki-67 index quantification. Only a few studies are currently in the literature that explore automated hot-spot detection^13,27–29^. Our pipeline generates Ki-67 indices that are comparable to the GS of exhaustive manual counting by a pathologist. Moreover, deep-SKIE automatically predicts a heatmap displaying background, non-tumor, G1 tumor, and G2 tumor, throughout the tissue, thereby providing a holistic view of tumor proliferation within the tissue. Additionally, since the double-immunostain technique is not standard, we integrated a cycle GAN architecture capable of transforming standard SS WSIs into DS WSIs. Our results confirm that the generated virtual DS images are comparable to the original adjacent DS counterparts. SKIE and deep-SKIE combined with cycle GAN improves the accuracy of Ki-67 index estimation and GI-NET grading as compared to the current clinical standard, while being significantly faster and more informative than existing methods.

On the technical front, SKIE differs from the typical pathologist’s protocol in determining the Ki-67 index in two major ways. First, SKIE relies on a widely available double-immunostain protocol that stains synaptophysin-expressing GI-NET regions with Permanent Red and Ki-67-positive nuclei brown with the traditional DAB chromogen. This double-immunostain allows SKIE to differentiate tumor from non-tumor cells using the Permanent Red channel. At the same time, this stain allows SKIE to quantify the Ki-67-positive tumor nuclei, which are presumed to be Ki-67 positive cells within the synaptophysin-positive tumor regions. In contrast, the current approach by pathologists is to use only a single IHC-stain for Ki-67 and rely on the pathologist’s assessment of whether an area consists of tumor or non-neoplastic cells. In some cases, this latter approach can present difficulties since the SS slide has limited information for accurate determination of tumor regions. A prior study has shown that the synaptophysin-Ki67 double-immunostain can significantly improve interobserver agreement for tumor grading among pathologists^10^. Another major difference is that SKIE uses the adjacent section stained with H&E to estimate the total number of tumor nuclei. The reason for this approach is that the Permanent Red stain can obscure hematoxylin counter-stained nuclei in the tumor regions, causing difficulty in automated quantitation of the total number of tumor nuclei. The adjacent H&E section provides superior contrast and thus a more accurate count of total tumor nuclei in the region of interest. Using the adjacent H&E section may cause slight differences in the total number of tumor nuclei within a tumor region. However, this limitation is likely a theoretical one given that typically adjacent sections from tumor specimens should not differ significantly in amount of tumor or total number of tumor cells.

SKIE uses a simplified algorithm to estimate the total number of tumor nuclei based on the median tumor nuclear size. Several nuclei segmentation algorithms are available in the literature^30^, including simple morphological analysis based methods to state-of-the art deep learning based approach^31^. However, segmentation performance always varies with image and tissue preparation variables, and till date there exists no robust, widely accepted nuclei segmentation algorithm for digital histology tissue images. The segmentation performance is especially poor when the nuclei are clumped together, as we have noticed as well in our dataset in this study. To circumvent this issue, we implemented an approximated method for nucleus count via computing the ratio between total area of the pixels in the image which were detected as nuclei and the average area of an individual nucleus. Our goal was to simplify the nuclei counting, while keeping the computational speed high.

The incorporation of cycle GANs further simplifies the pipeline by eliminating the requirement for DS sections, which may not gain wide use in histology laboratories. The pipeline allows the transformation of adjacent SS sections into DS sections, which were compared to the original DS WSI. Since these are adjacent sections, it is difficult to obtain a 1:1 correlation between the original and predicted DS images. Additionally, sections with artifacts (e.g. tissue folds, suboptimal stain, etc.) hampers the algorithm’s accuracy. Thus, it is necessary to have the input SS image stained optimally for the cycle GAN to function accurately. The virtual DS images when processed through SKIE and deep-SKIE, generated comparable results to the original DS images.

One limitation of SKIE is that this method cannot differentiate cell types within synaptophysin-positive tumor regions. It assumes all nuclei in tumor regions represent tumor cells. In cases where significant inflammatory cell infiltration is present within the tumor regions, SKIE will detect and include cells of this type into the Ki-67 calculation.

To the best of our knowledge, GI-NETs are almost 100% positive for synaptophysin. In our study of 50 cases, all were positive for synaptophysin. However, if a NET is negative for synaptophysin, then a different marker for neuroendocrine differentiation (e.g. chromogranin-A) could be used to identify tumor cells. As discussed above, we used a cycle GAN to create virtual DS WSIs that marked the NET regions. Therefore, one can use any marker for NET to train the cycle GAN algorithm to generate virtual DS images marking the tumor boundary on SS images or even H&E-stained images. Once the algorithm is trained sufficiently, this process eliminates the future need of synaptophysin (or chromogranin-A) to mark NET boundaries.

G3 GI-NETs were not included in this study because all tumors with a Ki-67 index of 20% or greater were specifically excluded, based on the prevailing definition of GI-NETs when the tumor samples were selected^10^. However, our algorithm would likely retain its accuracy in the subset of G3 GI-NETs because the morphologic properties of such tumors are similar to those of G1 and G2 GI-NETs. Since G3 GI-NETs are uncommon, currently we have insufficient number of cases to test, although this type of analysis is within the scope of future studies.

Our pipeline will be further optimized and integrated into a web-released software for public use with intuitive user interface. Additionally, the transfer of this technology into clinical practice would involve minimal costs as whole slide scanners are common in pathology laboratories, and the virtual staining process by cycle GANs would circumvent the need for any additional staining or imaging costs. The proposed framework will result in faster and more reproducible Ki-67 index quantification, thereby producing a more reliable tumor grading system.

By having a reliable automated algorithm for hot-spot detection and Ki-67 index quantification, it is possible in the future to explore in an empirical manner whether the current standard clinical practice as recommended by the WHO 2017 guidelines for GI-NET grading is optimal in terms of predicting patient prognosis and outcome. Given that any consensus guideline for clinical practice must conform to reproducible results among pathologists, we have been thus far restricted to approaches that take into account human limitation. For instance, the WHO 2017 and 2019 guidelines have set somewhat arbitrarily 500–2000 total tumor cells to be counted from the hot-spot for Ki-67 index quantification. With automation, such barriers are no longer obstacles and thus this “window” of total tumor cells analyzed can be varied or other methods can be tested. To this end, it would be possible to use computational analysis to compute the Ki-67 index from the whole slide rather than to be confined to limited hotspots. Ultimately, such methods need to be tested for their predictive and prognostic capabilities on patient cohorts with extensive follow-up clinical data.

## Supplementary information

Supplementary file1

## Data Availability

All of the source code used to derive the results presented within this study are made freely available at https://bit.ly/2UwQk3t. The biopsy data and additional explanation of the results, in the form of a supplemental document, are available at https://bit.ly/2X573gd.
