# Supplementary material for: Improving the accuracy of gastrointestinal neuroendocrine tumor grading with deep learning
Source: Sci Rep. 2020 Jul 6;10:11064. doi: 10.1038/s41598-020-67880-z (PMC7338406; doi:10.1038/s41598-020-67880-z)
Supplement: Supplementary file 1 — Supplementary file1 [file 41598_2020_67880_MOESM1_ESM.pdf]

SUPPLEMENTARY INFORMATION

Improving the accuracy of gastrointestinal neuroendocrine tumor grading with deep learning

Darshana Govind[1,†], Kuang-Yu Jen[2,†], Karen Matsukuma[2], Guofeng Gao[2], Kristin A. Olson[2], Dorina Gui[2], Gregory. E. Wilding[3], Samuel P. Border[1], Pinaki Sarder[1,*]

The State University of New York, Departments of [1]Pathology and Anatomical Sciences, and [3]Biostatistics, Buffalo, New York, United States
[2]University of California at Davis School of Medicine, Department of Pathology and Laboratory Medicine, Sacramento, California, United States

*Address all correspondence to: Pinaki Sarder, E-mail: pinakisa@buffalo.edu, Phone: (716) 829-2265, 955 main street, Buffalo, NY-14203

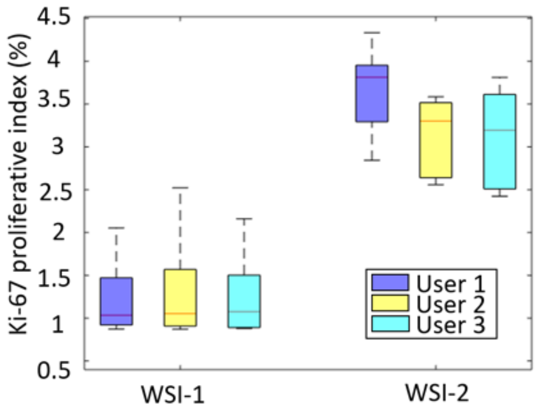

Supplementary Figure S1. **Ki-67 index based on inter- and intra-user variation in landmark selection.** Two cases with Ki-67 index between 3 ± 0.5% (i.e. on the border of G1 and G2) were analyzed to test the difference in Ki-67 index results based on variations in inter- and intra-user landmark selection. Plots represent Ki-67 indices for five candidate hot-spots for each set of user selected landmarks. User 2 and User 3 represent the same user who selected landmarks at two independent time points to asses intra-user variation. No change in tumor grade was observed based on variation in landmark selection.

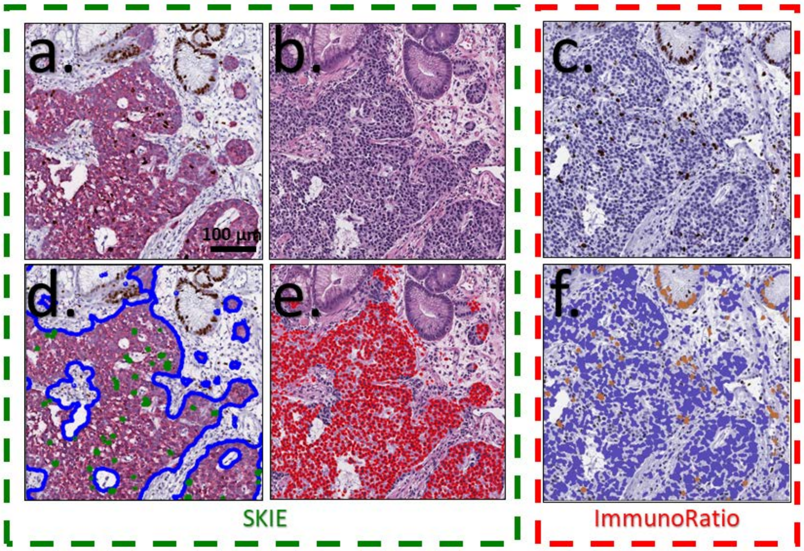

Supplementary Figure S2. **Comparison example of SKIE versus ImmunoRatio for the same field.** (a) Sample hot-spot from double-immunostained section. (b) Corresponding hot-spot from the adjacent H&E section. (c) Corresponding hot-spot from adjacent tissue section stained with Ki-67 and hematoxylin. (d) Image from S2a showing tumor region boundaries (blue) and Ki-67 positive cells (green) within tumor regions detected by SKIE. (e) Image from S2b showing nuclei (red) detected by SKIE within tumor boundaries in S2d. Ki-67 index of 2.59% was calculated by SKIE in this example. (f) Result of ImmunoRatio wherein the Ki-67 cells from non-neoplastic regions are included, thereby falsely elevating the proliferating index to 4.2% from the gold standard of 2.10%.

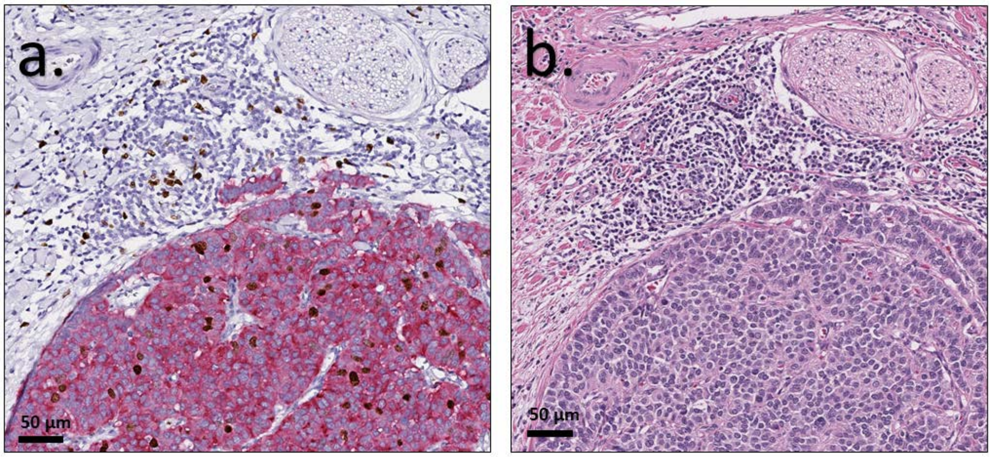

Supplementary Figure S3. Example of the same gastrointestinal neuroendocrine tumor focus. (a) Synaptophysin/Ki-67 double immunostain. (b) The adjacent hematoxylin and eosin-stained section. The double-immunostain shows synaptophysin (red) staining the tumor cells and Ki-67 (brown) staining the proliferating cell nuclei. Note the numerous Ki-67-positive inflammatory cells outside of the tumor focus that potentially could be erroneously included into the Ki-67 index calculation.

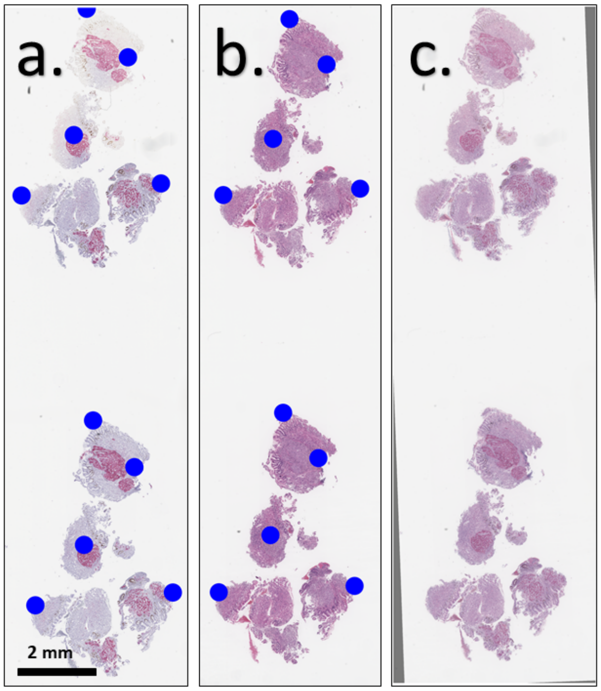

Supplementary Figure S4. Semi-automated image registration. Selection of 10 landmark points from each image. (a) Double-immunostained WSI with ten manually selected landmark points (shown in blue dots). (b) Adjacent H&E WSI with corresponding manually selected landmark points (shown in blue dots). (c) Overlay of warped H&E image with double-immunostained image.

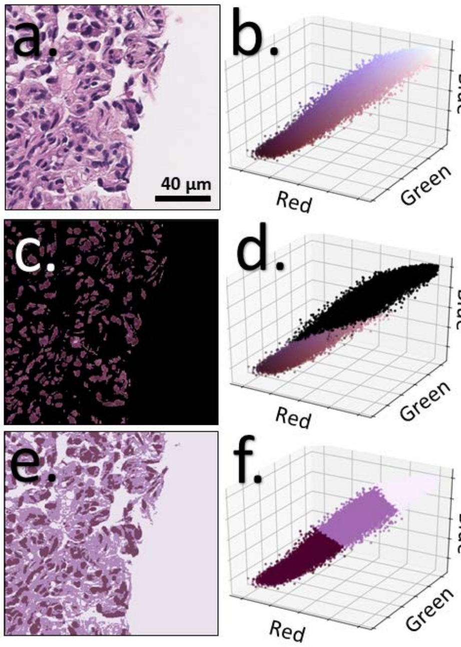

Supplementary Figure S5. **Unsupervised clustering of pixels to detect nuclei.** (a) Sample H&E image patch. (b) Color space of pixels in S5a. (c) Image of segmented nuclei with dark background. (d) Color space of pixel in S5c. (e) Image after k-means clustering with k = 3, representing hematoxylin, eosin and background. (f) Color space of pixels after k-means clustering.

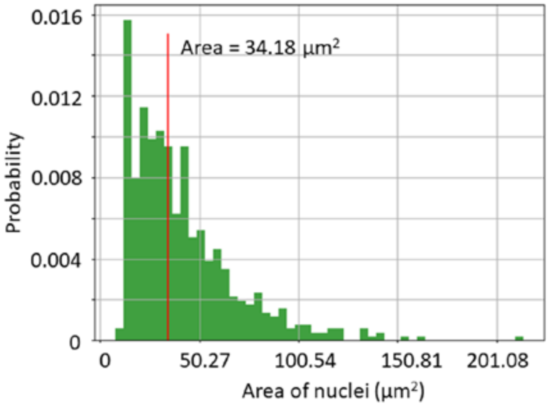

Supplementary Figure S6. **Estimation of single nucleus area.** The median of the distribution of all the single nuclei area is used to estimate the area of a single nucleus.

Feature	SKIE	ImmunoRatio
**Staining**	Ki-67, H&E, Synaptophysin	Ki-67
**Tumor region identification**	Yes	No
**Whole slide image analysis**	Yes	No
**Automated hotspot detection**	Yes	No
**Stain separation**	Color deconvolution based	Color deconvolution based
**Training samples**	None	None
**Nuclei segmentation**	Statistical estimation	Watershed
**Error rate against manual count**	0.84 ± 1.02%	2.15 ± 2.60%

Supplementary Table S1: Comparison of SKIE versus ImmunoRatio

Comparison	Observed kappa	Lower 95% CI	Upper 95% CI
Average Pathologist vs GS	0.67	0.38	0.96
SKIE vs GS (SKIE-picked HS)	0.62	0.32	0.91
SKIE vs GS (GS-picked HS)	0.73	0.38	1
ImmunoRatio vs GS (GS-picked HS)	0.16	0.0	0.40
Baseline vs GS	0	-0.83	0.83

Supplementary Table S2: Agreement statistics for SKIE and pathologists. The linear weighted Cohen's kappa, along with the upper and lower 95% confidence intervals (CI) are shown.

GS, gold standard; HS, hot spot.

Conditional probability of grade assignment	I (*n* = 45)	II (*n* = 5)
Pr(Average Pathologist|GS)	0.91 [0.79, 0.98]	1 [0.48, 1.0]
Pr(SKIE|GS); SKIE-picked HS	0.89 [0.76, 0.96]	1 [0.48, 1.0]
Pr(SKIE|GS); GS-picked HS	1 [0.92, 1]	0.6 [0.15, 0.95]
Pr(ImmunoRatio|GS); GS-picked HS	0.71 [0.56, 0.84]	0.6 [0.15, 0.95]

Supplementary Table S3: Conditional probability of class assignment.

Each value reported as: observed value [lower 95% confidence interval, upper 95% confidence interval].

GS, gold standard; HS, hot spot.
